# Surgical Mapping and Ablation in the Left Ventricular Summit Guided by Presurgery Pericardial Mapping

**DOI:** 10.19102/icrm.2019.100306

**Published:** 2019-03-15

**Authors:** Toshimasa Okabe, Gregory D. Rushing, Steven J. Kalbfleisch

**Affiliations:** ^1^Division of Cardiovascular Medicine, The Ohio State University Wexner Medical Center, Columbus, OH, USA; ^2^Division of Cardiothoracic Surgery, The Ohio State University Wexner Medical Center, Columbus, OH, USA

**Keywords:** Epicardial fat, epicardial mapping, left ventricular summit, premature ventricular complex, surgical ablation

## Abstract

Successful catheter ablation of ventricular arrhythmias arising from the left ventricular (LV) summit is challenging. The use of a catheter-based epicardial approach may be limited due to the proximity of the major coronary arteries and the presence of epicardial fat. Surgical cryoablation in the LV summit is a viable option for drug-refractory ventricular arrhythmias. Presurgical epicardial mapping can facilitate the surgical procedure by localizing the area of interest to allow for a more limited surgical dissection of the epicardial fat.

## Introduction

The left ventricular (LV) summit can be the location of idiopathic ventricular arrhythmias. The LV summit is the most superior portion of the epicardial LV outflow tract area bounded by the left anterior descending (LAD) and left circumflex (LCx) arteries and the great cardiac vein (GCV). Because of the technical challenge of reaching this area with a catheter, the proximity of the major coronary arteries bounding this region, and the presence of epicardial fat, the performance of catheter ablation within the LV summit can be a challenge and, in some patients, may be technically impossible to perform.^1^ In patients who cannot be ablated via a percutaneous epicardial catheter approach, surgical ablation has been performed. In many of these cases, extensive dissection of the epicardial fat around the coronary arteries, which is quite tedious and time-consuming, has been necessary to allow for the mapping and ablation of the premature ventricular contraction (PVC) focus. In the present case report, we describe the successful surgical ablation of frequent PVCs located close to the proximal LAD artery. The performance of the surgical technique and efficiency of the surgical ablation were facilitated by the presurgical epicardial catheter mapping, which localized the area of interest and enabled the surgeon to focus the dissection on a limited area for mapping and ablation.

## Case report

**Figure 1:**
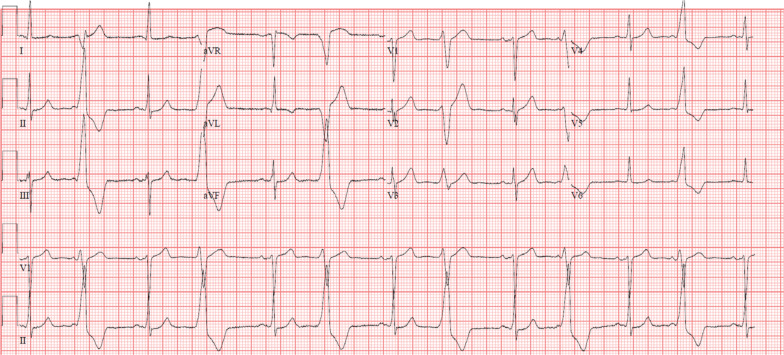
Twelve-lead ECG shows sinus rhythm with ventricular bigeminy.

A 60-year-old male with a medical history of hypertension and obesity (body mass index: 36 kg/m^2^) presented with frequent PVCs and cardiomyopathy. There was no family history of cardiomyopathy or sudden cardiac death. A 12-lead electrocardiogram (ECG) showed sinus rhythm with ventricular bigeminy. The PVC morphology was a left bundle branch pattern with inferior axis **(Figure 1)**. Holter monitoring showed monomorphic PVCs and repetitive nonsustained ventricular tachycardia (VT) with a total ventricular ectopy burden of 40%. Cardiac magnetic resonance (CMR) imaging demonstrated globally hypokinetic and dilated LV with an LV ejection fraction (EF) of 35% to 40% and limited midmyocardial to epicardial fibrosis in the basal inferolateral wall. There was no evidence of myocardial inflammation or edema. Coronary angiogram revealed nonobstructive coronary artery disease. The patient’s PVC burden was considered to be contributing to his cardiomyopathy, and he was referred for a PVC ablation. Additionally, given the nonischemic pattern of myocardial fibrosis on CMR, right ventricular (RV) electrogram-guided endomyocardial biopsy (EMB) was recommended to rule out an early manifestation of inflammatory or infiltrative cardiomyopathies. The first procedure consisted of endocardial mapping of the RV outflow tract (RVOT), LV outflow tract (LVOT), and aortic sinuses of Valsalva as well as epicardial mapping via the GCV. The earliest ventricular activations in the RVOT, GCV, and LVOT were 24 ms, 40 ms, and 45 ms later than the QRS onset on the 12-lead ECG, respectively. There was no site with presystolic ventricular activation, and no ablation was performed. Programmed ventricular stimulation did not induce sustained VT. Detailed mapping of the RV endocardium using a 3.5-mm irrigated-tip catheter (ThermoCool^®^ SF; Biosense Webster, Diamond Bar, CA, USA) showed a normal bipolar electrogram voltage of greater than 1.5 mV, and EMB was not performed. Metoprolol and mexiletine were initiated but were not effective in suppressing the PVCs.

**Figure 2:**
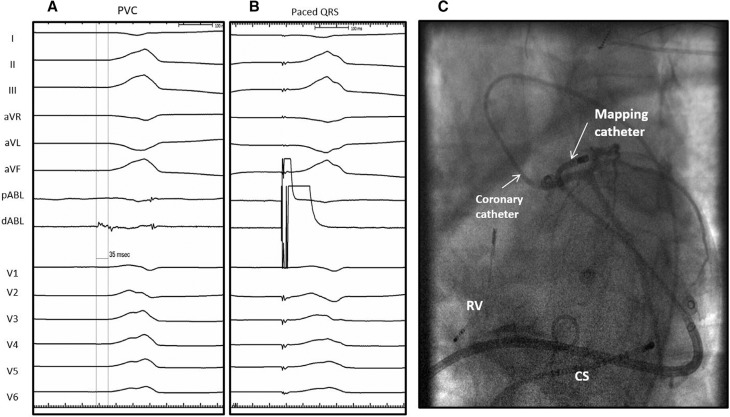
**A:** Ventricular activation (dABL) precedes the onset of surface QRS by 35 ms. pABL and dABL denote the proximal and distal bipolar electrode pairs of the mapping catheter, respectively. **B:** Pacing at this location showed a 10/12 ECG QRS match. **C:** Coronary angiogram in the left anterior oblique caudal projection. Mapping catheter was placed in the pericardial space through a deflectable sheath. The earliest activation site is located just medial (septal) to the proximal LAD artery. RV: quadripolar catheter in the right ventricular apex; CS: decapolar catheter in the coronary sinus.

Because of persistent ventricular ectopy and decreased LV function despite medical therapy, the patient was referred for epicardial mapping and possible ablation. Under general anesthesia, percutaneous epicardial access was obtained via a subxiphoid approach using a needle-in-needle technique.^2^ The earliest activation (−35 ms pre-QRS) was localized to the anterobasal LV area. Pacemapping from this location produced a 10/12 ECG QRS match. Coronary angiogram in the LAO projection demonstrated that the mapping catheter tip at this site was located less than 5 mm medial to the proximal LAD artery **(Figure 2)**. Owing to the proximity of the best mapping site to the LAD artery and the concerns of coronary artery injury, no ablation was performed, and the patient was started on amiodarone.

Repeat Holter monitoring continued to show a high PVC burden (45%) despite amiodarone and metoprolol. Given the persistent drug-refractory PVCs and associated cardiomyopathy, the patient was referred for a surgical PVC ablation. It was hoped that direct visualization of the LV summit would allow for surgical dissection of the epicardial fat around the LAD artery, give more precise localization of the PVC origin, and ensure safe and effective energy delivery. Before the operation, he underwent repeat coronary angiogram to reassess for coronary artery disease. The proximal LAD artery stenosis was determined to be flow-limiting, as assessed by fractional flow reserve. Consequently, the patient was offered the option of surgical PVC ablation and concomitant single-vessel coronary artery bypass grafting via a median sternotomy approach, which was to be performed in a hybrid electrophysiology laboratory.

The presurgical procedure setup was planned out to allow for placement of modified 12-lead ECG and CARTO^®^ patches (Biosense Webster, Diamond Bar, CA, USA) during the performance of the median sternotomy. The procedure took place 12 months after the last percutaneous epicardial procedure. The precordial ECG and CARTO^®^ patches (Biosense Webster, Diamond Bar, CA, USA) were all placed laterally to the left nipple line to allow for a sufficiently large surgical field to be realized. Given the more lateral displacement of the precordial ECG leads, a 12-lead PVC morphology template was obtained only after the placement of a sternal retractor to allow for pacemapping if needed. After completing the median sternotomy, the left internal mammary artery (LIMA) was harvested in a pedicle fashion. The LV summit was approached for the purpose of mapping the PVC focus. The initial plan was to perform mapping without cardiopulmonary bypass (CPB) because of concerns regarding that unloading the LV would result in PVC suppression. Within the chest cavity, the heart was rotated to the left and required a significant amount of torsion to expose the posteriorly displaced LV summit. This manipulation led to hemodynamic instability and repetitive nonclinical ventricular ectopy. The patient was therefore given a CPB pump during cardiac manipulation and PVC mapping. The clinical PVCs persisted following the initiation of CPB.

**Figure 3:**
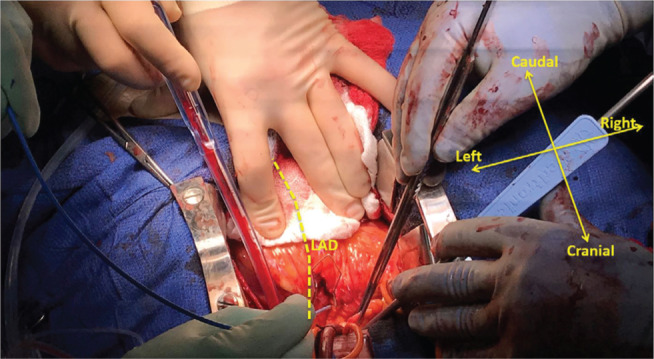
The epicardial fat over the LV anterobasal area was dissected and a small retractor was placed in the epicardial fat to expose the underlying myocardial surface. A mapping catheter was located just medial (septal) to the proximal LAD artery.

**Figure 4:**
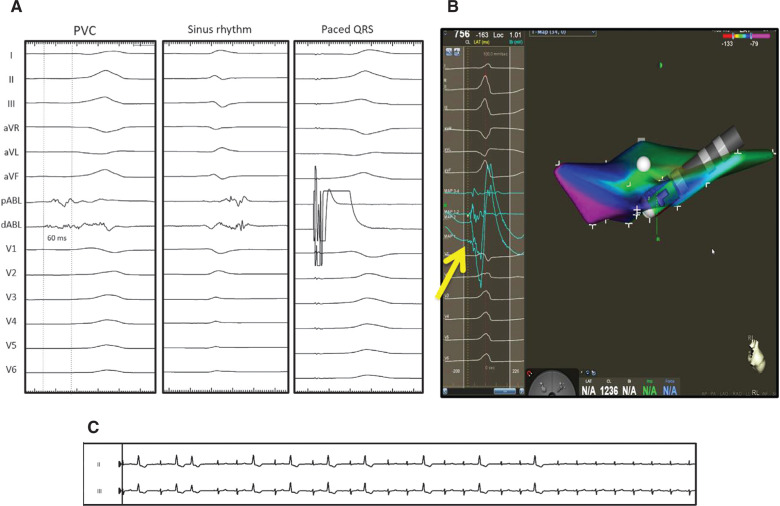
**A:** On the left, ventricular activation (dABL) precedes the onset of surface QRS by 60 ms. pABL and dABL denote the proximal and distal bipolar electrode pairs of the mapping catheter, respectively. In the middle, an abnormal electrogram (low-amplitude, fractionated, and long-duration) is notable in sinus rhythm. On the right, pacing at this location produced a nearly perfect QRS match. **B:** Three-dimensional electroanatomic mapping shows the catheter location at this location. The unipolar electrogram at this site showed a QS signal with the PVC (arrow). **C:** PVC was suppressed within 20 seconds of cryoenergy application.

After adequate exposure of the LV summit, a thick layer of epicardial fat (estimated thickness: 5 mm) overlying the proximal coronary circulation was noted. The left main coronary artery and proximal LAD artery were visualized and, based on the presurgical mapping information, the epicardial fat just medial to the proximal LAD artery was surgically dissected parallel to the LAD artery to expose the underlying myocardium. The epicardial surface in this region was then mapped using a 3.5-mm irrigated-tip catheter (ThermoCool^®^ SF; Biosense Webster, Diamond Bar, CA, USA) and CARTO^®^ (Biosense Webster, Diamond Bar, CA, USA) **(Figure 3)**. A very early activation site (−60 ms pre-QRS PVC) was identified on the epicardium, and any slight movement away from this location in all directions resulted in a later activation time. The unipolar electrogram at this site showed a QS signal with the PVC, and there was a long-duration fractionated electrogram seen with the sinus rhythm beats. Pacemapping from this location produced a nearly perfect QRS match **(Figure 4)**. Cryoablation was performed at this location with the cryoICE ablation system (CRYO2; AtriCure, Mason, OH, USA). Approximately 3 cm of the distal potion of the malleable aluminum probe (total probe length: 10 cm) was exposed and placed parallel to the proximal LAD artery just outside the LV summit. The cryoprobe was set to achieve a temperature of −65°C for three minutes and, within 20 seconds of the cryoablation initiation, the PVCs were suppressed **(Figure 4)**. Occasional PVCs returned after thawing. Another five-minute cryoablation application at the same settings and to the same location was completed and resulted in complete PVC suppression. During the second application, transient anterior ST-segment elevation was seen on the surface ECG recording, which resolved quickly after thawing. The patient then underwent uncomplicated, pump-assisted, beating-heart LIMA–LAD artery bypass grafting. There were no acute complications or PVCs on telemetry during the remainder of the hospitalization. He developed postoperative atrial fibrillation, which required a four-week course of amiodarone for suppression. A 24-hour Holter monitoring session performed at three months after the ablation, off antiarrhythmic therapy, showed a PVC burden of less than 0.1%, whereas the echocardiogram demonstrated normalization of the LVEF.

## Discussion

The performance of catheter ablation of ventricular arrhythmias arising from the epicardial aspect of the anterobasal LV is challenging. Specifically, the area known as the LV summit, defined as the portion of the epicardium bounded by the LAD artery, LCx artery, and GCV, can be particularly difficult to ablate within. In some patients, this region may be inaccessible with a catheter placed in the pericardial space, whereas, in others, even if a catheter can reach this area, safe and effective energy delivery may be unattainable due to proximity of major coronary arteries and a thick layer of epicardial fat.^3,4^ Various techniques have been described to overcome challenges associated with the mapping and ablation of arrhythmias arising from the LV summit. These include mapping and ablation from adjacent structures such as the RVOT, LVOT, GCV, and left atrial appendage; coronary venous ethanol injection; and intramyocardial needle ablation.^5–8^

In our attempted percutaneous epicardial ablation procedure, the earliest activation site was localized just medial (septal) to the proximal LAD artery, and no ablation was performed due to safety concerns. Based upon the findings at the time of the surgical procedure, it appears that, even if radiofrequency energy had been delivered in this location, PVC elimination would have been unlikely due to the thick layer of epicardial fat overlying this region.

The real key to the success of this procedure was the multidisciplinary approach applied in combination with the use of a hybrid electrophysiology laboratory. The hybrid electrophysiology laboratory is a newly designed procedure room that allows for open-chest surgical procedures to be performed within the electrophysiology procedure area. It combines the advanced fluoroscopic imaging and mapping technology of the electrophysiology laboratory with the operating room equipment and sterility standards of a cardiovascular operating room. The presurgical pericardial mapping, which included the creation of a three-dimensional electroanatomic activation map coupled with coronary angiography, was indispensable in successfully guiding and limiting the amount of epicardial fat dissection required. Cryoablation as the energy source was specifically chosen in this case due to the proximity of the proximal LAD artery in the hope of avoiding direct thermal injury or spasm of the proximal coronary artery.

A few other crucial factors that contributed to the success of this case should be noted. Although mapping was performed under direct visualization, the catheter tip was effectively buried in the thick pericardial fat through the small window created by surgical dissection, rendering the operator essentially blind to the actual location of the catheter tip. The use of a three-dimensional electroanatomic mapping system (CARTO^®^; Biosense Webster, Diamond Bar, CA, USA) greatly assisted in localizing the catheter tip in this small space. As previously described, placement of 12-lead ECG and CARTO^®^ patches (Biosense Webster, Diamond Bar, CA, USA) were modified to allow for the achievement of a sufficiently large sterile surgical field. The lateral displacement of ECG leads accounts for the different QRS morphology of clinical PVCs seen during surgical mapping as compared with the QRS morphology seen on the standard 12-lead ECG. There was no significant magnetic interference with the CARTO^®^ mapping system (Biosense Webster, Diamond Bar, CA, USA) despite the use of a metal sternal retractor. Although a minimally invasive surgical approach for mapping and ablation using a robotic surgical system has been reported, in this case, the strikingly thick epicardial fat and the need for concomitant coronary revascularization necessitated direct visualization via median sternotomy.^9,10^

The successful ablation site showed several notable findings. First, after the dissection of the epicardial fat, the earliest timing (−60 ms pre-QRS) directly on the epicardium was 25 ms earlier than the earliest activation timing recorded during the percutaneous mapping over the epicardial fat. The bipolar electrogram after removal of the epicardial fat also exhibited more prominent electrogram fractionation and continuous and longer electrogram duration, which are the findings consistent with “near-field” recording of the arrhythmogenic tissue^11^ **(Figures 2A and 4A)**. Direct contact with the myocardium surface likely contributed to the localization of the true origin of the arrhythmia. Second, despite the absence of myocardial fibrosis in the LV anterobasal wall on gadolinium-enhanced CMR, an abnormal electrogram characterized by low amplitude, fractionation, and long duration was observed in sinus rhythm, indicating an underlying abnormal arrhythmogenic substrate in this location.

The safety and efficacy of surgical ablation of ventricular arrhythmias arising from the LV summit have not been well-established to date. There are only a few case reports and small case series available in the literature.^12–14^ Technically, the successful ablation site in our case was adjacent to the proximal LAD artery, just outside the LV summit. However, the same concerns and limitations are applicable, and we believe that the present case adds to the limited literature on surgical mapping and ablation in the LV summit.

## Conclusion

Surgical cryoablation in the LV summit is a viable option for the treatment of drug-refractory ventricular arrhythmias. Presurgical epicardial mapping can help to facilitate the surgical procedure by localizing the area of interest to allow for a more limited surgical dissection to reach the epicardial surface for mapping and ablation.
